# Perspectives on cancer therapy—synthetic lethal precision medicine strategies, molecular mechanisms, therapeutic targets and current technical challenges

**DOI:** 10.1038/s41420-025-02418-8

**Published:** 2025-04-16

**Authors:** Shixuan Peng, Mengle Long, Qisheng Chen, Zhijian Yin, Chang Zeng, Wanyong Zhang, Qingyang Wen, Xinwen Zhang, Weiqi Ke, Yongjun Wu

**Affiliations:** 1 Department of Oncology, Graduate Collaborative Training Base of The First People’s Hospital of Xiangtan City, Hengyang Medical School, University of South China, Hengyang, Hunan 421001 China; 2 Department of Oncology, The First People’s Hospital of Xiangtan City, Xiangtan, Hunan 411101 China; 3 Department of Anesthesiology, The First People’s Hospital of Chenzhou, The Chenzhou Affiliated Hospital, Hengyang Medical School, University of South China, Chenzhou, Hunan 423000 China; 4 Department of Pathology, Yueyang Central Hospital, Yueyang, China; 5 Department of Pathology, Xianning Central Hospital, The First Affiliated Hospital of Hubei University of Science and Technology, Xianning, 437100 Hubei China; 6 Department of Anesthesiology, The First Affiliated Hospital of Shantou University Medical College, Shantou, Guangdong Province China; 7 Department of Pathology, Xiangtan Center Hospital, Xiangtan City, Hunan province 411100 China; 8 Department of Pathology, The Affiliated Hospital of Hunan University, Xiangtan City, Hunan Province China

**Keywords:** Cancer epigenetics, Cancer epigenetics

## Abstract

In recent years, synthetic lethality has become an important theme in the field of targeted cancer therapy. Synthetic lethality refers to simultaneous defects in two or more genes leading to cell death, whereas defects in any single gene do not lead to cell death. Taking advantage of the genetic vulnerability that exists within cancer cells, it theoretically has no negative impact on healthy cells and has fewer side effects than non-specific chemotherapy. Currently, targeted cancer therapies focus on inhibiting key pathways in cancer. However, it has been found that over-activation of oncogenic-related signaling pathways can also induce cancer cell death, which is a major breakthrough in the new field of targeted therapies. In this review, we summarize the conventional gene targets in synthetic lethality (PARP, ATR, ATM, WEE1, PRMT) and provide an in-depth analysis of their latest potential mechanisms. We explore the impact of over-activation of pathways such as PI3K/AKT, MAPK, and WNT on cancer cell survival, and present the technical challenges of current research. Important theoretical foundations and insights are provided for the application of synthetic lethal strategies in cancer therapy, as well as future research directions.

## Facts

- Mechanisms of acquired resistance to PARP inhibitors: Understanding how cancer cells become resistant to PARP inhibitors, particularly in BRCA-mutated malignancies, is critical. Research could focus on the genetic and epigenetic modifications that cause resistance and how to overcome them.
- Optimizing Combination Therapies: The publication discusses the possibilities for integrating synthetic lethality methods with other treatments, such as immunotherapy. Future research could look into the most effective pharmacological combinations and sequencing for improving therapeutic outcomes while reducing side effects.
- Biomarker Discovery and Validation: Finding predictive biomarkers for synthetic lethality therapy is critical for personalised medicine. More research is needed to identify and confirm biomarkers that can reliably predict patient response to specific synthetic lethality treatments.
- Exploring Synthetic Lethality in Different Cancer Types: While synthetic lethality has been extensively examined in some cancer types, such as ovarian and breast cancer, its promise in others remains largely untapped. Future research could concentrate on developing synthetic lethality solutions for a larger range of malignancies.
- Technical Challenges in Synthetic Lethality Research: This document discusses the difficulties in identifying synthetic lethal partner genes and developing drugs. Future research could focus on developing new technologies and ways to tackle these issues, such as better computational tools for genetic analysis and novel medication delivery systems.

## Open questions

- How can we accurately predict which cancer patients will respond to synthetic lethality treatments, and which will develop resistance?
- What are the long-term effects of synthetic lethality treatments on cancer cells and the patient’s overall health?
- How can we overcome the acquired resistance observed in cancer cells treated with synthetic lethality strategies, such as PARP inhibitors?
- What is the role of the tumor microenvironment in the success or failure of synthetic lethality treatments?
- Can synthetic lethality be effectively combined with immunotherapies to improve patient outcomes?
- How do genetic and epigenetic heterogeneities within tumors affect the success of synthetic lethality treatments?
- What new synthetic lethal pairs can be identified and targeted in cancer cells, especially in cancer types where traditional targets are less prevalent?
- How can we use advanced computational methods and artificial intelligence to better understand synthetic lethality and identify new therapeutic targets?

## Introduction

Cancer is a group of diseases that originate in different organs or tissues and are characterized by the abnormal proliferation of cells that are out of control. These abnormal cells can break through normal boundaries, invade neighboring areas and potentially metastasize to distant organs [1]. As cancer has a complex evolutionary trajectory, it has become a highly malignant disease worldwide, accompanied by significant morbidity and mortality. Traditional treatments such as surgery, chemotherapy, and radiotherapy have limitations in killing cancer cells but also damaging the surrounding normal tissues, especially causing irreversible damage to radiosensitive tissues and organs [2, 3]. In order to improve the prognosis of cancer patients, innovations in treatment modalities have become imminent. Nowadays, targeted therapies have become the latest therapeutic strategy for many cancer types, and the strategy based on the principle of synthetic lethality is particularly notable. The core principle of this strategy is to inhibit key gene targets in the signaling pathway according to the patient’s genome characteristics, so as to cause the cell cycle of the cancer cells to stagnate or to die, and thus achieve the therapeutic purpose [4–6]. Compared to the “one-size-fits-all” approach of the past, the shift to personalized treatment based on patient-specific biomarkers allows for the precise targeting of specific cancer cell defects with less impact on normal cells and fewer side effects [7]. This promising anti-cancer treatment strategy is expected to become the mainstream of clinical treatment in the future, bringing better treatment results and quality of life to cancer patients.

**Fig. 1:**
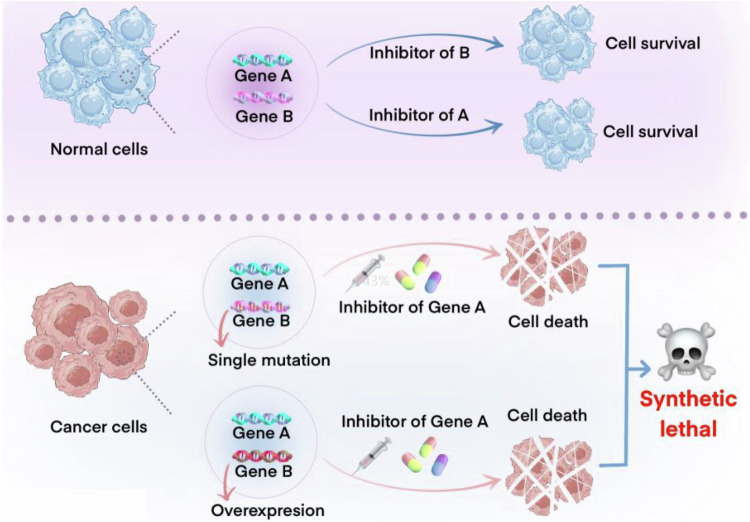
The concept of synthetic lethality. Individual inhibition or deletion events of gene A or B are compatible with cell viability (upper part). The inhibition or defects of two genes (A and B) in cancer cells lead to cell death (lower part).

As early as the early 1920s, the American geneticist Calvin Bridges found incompatibility between allele pairs in Drosophila melanogaster crosses, where the non-alleles could cause cell death only in combination [8]. Subsequently, Theodore Dobzhansk observed the same result in Drosophila pseudobscura and formally proposed the concept of “synthetic lethality” to describe the complementary lethality between different genes [9, 10]. Synthetic lethality (SL) is the phenomenon whereby a defect in one gene alone does not cause cell death, but when combined with a defect in another specific gene, i.e., multiple defects in two or more related genes occur at the same time, it leads to specific cell death or apoptosis, whereas the occurrence of a single defect in one of the genes concerned is tolerated for the survival of the cell [11, 12] (Fig. 1). As scientists studied synthetic lethality more intensively, they began to systematically classify the phenomenon into two broad categories: unconditional (or primitive) synthetic lethality and conditional synthetic lethality [13].

SL can inhibit specific genes to disrupt the DNA repair function of cancer cells by interfering with the DNA damage response (DDR) signaling pathway [14]. There are countless targeted drugs available today to inhibit oncogenic signaling with remarkable efficacy. Unfortunately, the long-term use of targeted drugs can lead to acquired resistance in some cancer cells, making long-term control of advanced cancers with these drugs difficult to achieve [15]. However, studies have found that over-activation of oncogenic signaling pathways can also disrupt cancer homeostasis and lead to death, reversing the conventional wisdom that inhibition of oncogenic signaling pathways is required to treat cancer. For example, cMYC aberrant expression is closely associated with a variety of tumors, and its overexpression makes cancer cells more sensitive to apoptosis induced by various stimuli. Pharmacological upregulation of β-catenin and cMYC through GSK-3β inhibition triggers RAS-driven apoptosis in cancer cells and inhibits tumor growth [16–18]. This implies that cancer cells may only survive under specific signaling conditions, and that when these conditions are manipulated, the effects on cancer cells become more pronounced [19]. This finding has attracted widespread attention from cancer biologists as it provides a new perspective on cancer treatment.

## DNA SSBs and DNA DSBs-related synthetic lethal major pathways and targets

**Fig. 2:**
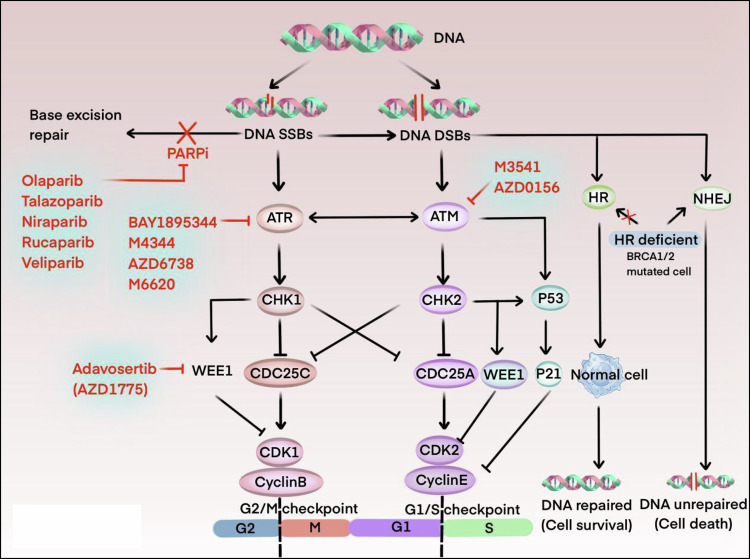
DNA SSBs and DNA DSBs-related synthetic lethal major pathways and targets. The repair of DNA double-strand breaks (DSBs) is mainly done through rapid non-homologous end joining (NHEJ) and slow but high-fidelity homologous recombination (HR). ATM is activated when double strand breaks, while ATR is activated after replication stress leads to single-strand breaks (SSB). The main downstream targets of ATM include CHK2 and P53, which regulate cell cycle block or promote cell apoptosis. The main target of ATR is CHK1, which leads to phosphorylation and inactivation of CDC25A and CDC25C, which inhibits the activation of CDK2 and CDK1 and prevents cells from entering S phase and M phase. Wee1 directly regulates the activity of CDKs in S and G2 phases, similar to the role of CHK1 in cell cycle regulation.

DDR is responsible for detecting and repairing different types of DNA damage and blocking the cell cycle, thus providing accurate DNA repair and promoting aberrant apoptosis to increase genome stability [20–22]. The main pathways of DNA damage repair include base excision repair (BER), homologous recombination (HR), non-homologous end connection (NHEJ), nucleotide excision repair (NER) and mismatch repair (MMR) [23]. They are coordinated mainly by two different kinase signaling cascade reactions, regulating the DNA replication stress checkpoint in M and G2 phases by interfering with the ATR-CHK1-WEE 1 pathway, and regulating the DNA replication stress checkpoint in S and G2 phases by the ATM- CHK2TP53 pathway, which regulates the DNA stress checkpoint in S and G1 phases, induces synthetic lethality in cells such as repressor TP53, ARID 1 A, or ATM [24–27] (Fig. 2). A large number of studies have described the discovery and development of synthetic lethal gene targets and revealed their inhibitor interactions, and this paper summarizes the molecular mechanisms and clinical applications of conventional gene targets and their inhibitors, aiming to inform further research and clinical practice. Through a deeper understanding of these mechanisms, more effective therapeutic strategies can be developed in the future to enhance the prognosis and quality of life of cancer patients.

### PARP inhibitors

Poly (ADP-ribose) polymerase 1 (PARP1), one of the primary members of the PARP family, is a crucial DNA repair enzyme in the base excision repair (BER) pathway [28]. Nearly 80% of the poly-ADP-ribosylated (PAR) strands are synthesized in the DNA repair pathway, and thus PARP 1 is the primary target of PARPis in clinical trials [29]. PARP inhibitors (PARPi) cause the accumulation of unrepaired single-strand breaks (SSBs) primarily by blocking the BER pathway, when DNA replication forks stall and collapse at persistent SSB lesions, ultimately induces lethal DNA double-strand breaks (DSBs) [30–32]. PARPi also induces HR-deficient (HRD) tumor death by physically trapping at DNA break sites using a PARP trapping mechanism in conjunction with the PARP 1 enzyme on chromatin. In normal cells, genomic instability caused by DNA double-strand breaks is accurately repaired by homologous recombination (HR) in the form of sister chromatids and is largely unaffected by PARPi [33]. In contrast, cells with HR-deficient BRCA 1/2 mutations, in which DNA DSBs cannot be repaired in a timely manner in the error-prone non-homologous end joining NHEJ (nonhomologous end joining) pathway, are selectively targeted by PARPi to trigger cell death [34–37] (Fig. 2).

PARP inhibitors were the first drugs to achieve success based on the concept of synthetic lethality. In 2014, the first FDA-approved PARPi Orapanil was used to treat women with metastatic ovarian cancer who had received three or more lines of chemotherapy, and maintenance therapy for women with ovarian cancer mutations in BRCA1/2 who achieved complete or partial remission after platinum chemotherapy [38–40]. Subsequently, Niraparib and Rucaparib were approved for the treatment of ovarian cancer [41, 42]. Today, the use of PARP inhibitor orapanil is expanding from ovarian cancer therapy to other tumor types with homologous recombination defects, such as breast, prostate and pancreatic cancer [43–46]. In addition to Orapanil, FDA also approved PARP inhibitors such as Nilaparib, Rucaparib and Tazemetostat for the corresponding cancer treatment [47–49](Table 1).

**Table 1:** PARP, ATR, ATM, WEE1 and PRMT inhibitors in the field of synthetic lethality: monotherapy and combination therapy.

Drug target	Cellular process and mechanism	Drug	Intervention	Gene/ Biomarkers	Cancer Type	Clinical trail phase	NCT number	Ref
**PARP**	Regulate cell proliferation and differentiation; repair DNA single-strand and double-strand breaks	Olaparib	Monotherapy	BRCA	Ovarian cancer	III	NCT01844986	[176]
				BRCA1/2	Breast cancer	III	NCT02032823	[177]
				BRCA	Pancreatic cancer	III	NCT02184195	[44]
				HRR	Prostate cancer	III	NCT02987543	[178]
			Olaparib + Abiraterone	BRCA1/2	Prostate cancer	III	NCT03732820	[179]
			Olaparib + Paclitaxel	ATM	Gastric cancer	III	NCT01924533	[180]
		Talazoparib	Monotherapy	BRCA	Breast cancer	III	NCT01945775	[181]
		Niraparib	Monotherapy	BRCA	Ovarian cancer	III	NCT01847274	[182]
			Niraparib + Abiraterone + prednisone	HR	Prostate cancer	III	NCT03748641	[183]
		Rucaparib	Monotherapy	BRCA	Prostate cancer	II	NCT02952534	[184]
				BRCA1/2	Ovarian cancer	III	NCT02855944	[185]
			Rucaparib + Nivolumab	-	Ovarian cancer	III	NCT03522246	[186]
		Veliparib	Veliparib + carboplatin + paclitaxel	BRCA1/2	Metastatic breast cancer	III	NCT02163694	[187]
		Stenoparib	Monotherapy	-	Advanced Ovarian Cancer, Metastatic Breast Cancer	II	NCT03878849 NCT03562832	No publications
		Venadaparib	Monotherapy	-	Homologous RecombinationDeficiency, Solid Tumors	II	NCT04174716	
		Amelparib	Monotherapy	-	Acute Ischemic Stroke	II	NCT03062397	
		SC10914	Monotherapy	BRCA1/2	Breast Cancer Metastatic	II	NCT04556292	No publication
		AMXI-5001	Monotherapy	-	Advanced Malignant Neoplasm, Breast Cancer, Ovarian Cancer	I/II	NCT04503265	No publication
		saruparib (AZD5305)	New hormonal agents	-	Metastatic Prostate Cancer	II	NCT05367440	No publication
**ATR**	DNA stress activates signal transduction at cell cycle checkpoints and triggers ATR-mediated DNA damage sensing	Ceralasertib (AZD6738)	Monotherapy	TP53	AST, LC, gastric cancer, breast cancer	I/II	NCT02264678	[188]
			AZD6738 + Durvalumab	DDR defect	NSCLC	II	NCT03334617	[189]
			AZD6738 + Radiotherapy	TP53	AST	I	NCT02223923	[190]
		BAY1895344	Monotherapy	DDR defect	AST and lymphoma	1	NCT03188965	[191]
			BAY1895344 + Niraparib	TP53	Ovarian cancer	I	NCT04267939	[188]
		M4344	Monotherapy or carboplatin	ATM、ARID1A、DAXX	AST	I	NCT02278250	[192]
			M4344 + Niraparib	TP53	Ovarian cancer	1	NCT04149145	[193]
			Berzosertib + Topotecan (Hydrochloride)	TP53	LC	I/II	NCT02487095	[194]
		Berzosertib	Veliparib or cisplatin	-	Solid tumors	I	NCT02723864	
			Avelumab	DDR gene mutations	Solid tumors	I/II	NCT04266912	
		RP-3500	Olaparib	TP53, ATM, SF3B1, XPO1 or POT1 mutations	CLL	I/II	NCT05405309	
		ATRN-119	Monotherapy	DDR gene mutations	AST	I/II	NCT04905914	
		ART-0380	Monotherapy, gemcitabine or irinotecan	ATM	AST, MST	I/II	NCT04657068	
**ATM**	Activate cell cycle checkpoints; identify damaged DNA and trigger ATM-mediated DNA damage response pathway to repair damaged DNA chains	M3541	Monotherapy	DSB	Solid tumor	I	NCT03225105	[195]
		AZD0156	Monotherapy, olaparib, irinotecan or FOLFIRI	-	Solid tumor	I	NCT02588105	[196]
		AZD1390	Radiotherapy	-	Recurrent Glioblastoma Multiforme	I	NCT03423628	No publication
		Lartesertib(M4076)	Monotherapy	-	AST	I	NCT04882917	
		XRD-0394	Radiotherapy (palliative)	-	AST, MST, RST	I	NCT05002140	
**WEE1**	Serine-threonine protein kinase; regulation of G2max M checkpoint by CDC-2 inhibition.	Adavosertib (AZD1775)	Monotherapy	TP53	Solid tumor	I	NCT01748825	[197]
			Adavosertib + Gemcitabine+Cisplatin + Carboplatin	TP53	AST	I	NCT00648648	[198]
			Adavosertib + Radiotherapy+ gemcitabine	TP53	Pancreatic cancer	I	NCT02037230	[199]
			AZD1775 + irinotecan	RAS/BRAF	Colorectal cancer	I	NCT02906059	[200]
		IMP-7068	Monotherapy	-	AST	I	NCT04768868	
		ZN-c3	Monotherapy	-	High-Grade Serous Ovarian	II	NCT05128825	No publication
			Monotherapy, Niraparib	-	Uterine Serous Carcinoma, Ovarian Cancer	I/II	NCT05198804	
**PRMT**	Substrate binding pocket and extra contact with SAM binding pocket	GSK3326595	Monotherapy+ pembrolizumab	PD1	AST and lymphoma	I	NCT02783300	[201]
		AMG193	Monotherapy	MTAP	MTAP-null solid tumors	I/II	NCT05094336	No publications
		SCR-6920	Monotherapy	-	Solid tumor, Non-HodgkinLymphoma	I	NCT05528055	No publications
		PRT543	Monotherapy	-	AST	I	NCT03886831	[202]
		MRTX1719	Monotherapy	MTAP	NSCLC, AST, Pancreatic cancer	I/II	NCT05245500	[203]
		TNG908	Monotherapy	MTAP	MTAP-null solid tumors	I/II	NCT05275478	No publications

*NSCLC* Non-small cell lung cancer, *AST* Advanced solid tumor, *LC* lung cancer, *DDR* DNA damage response, *MST* metastatic solid tumor, *RST* refractory solid tumor, *CLL* chronic lymphocytic leukaemi.

**Fig. 3:**
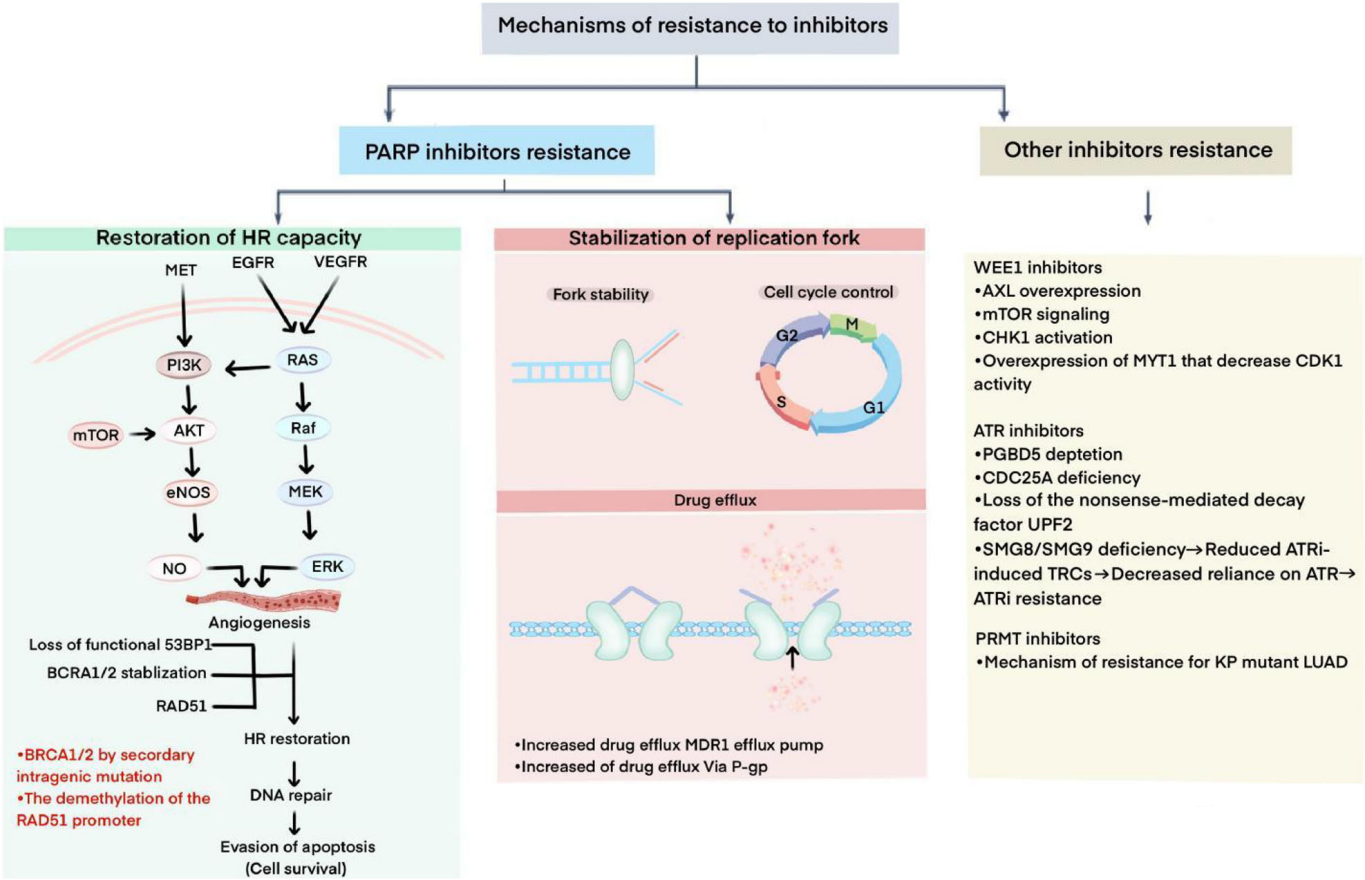
Mechanisms of resistance to inhibitors. The resistance of cancer to inhibitors can be innate or acquired. The underlying mechanisms of drug resistance are varied and often multifactorial. For PARP inhibitors (PARPi), the most common drug resistance mechanisms include functional recovery of homologous recombination (HR) genes such as BRCA and RAD51, enhanced replication bifurcation stability leading to DNA repair and replication bifurcation restart, the role of drug efflux pumps, and changes in tumor microenvironment leading to impaired drug delivery.

As with most synthetic lethal inhibitors, the clinical therapeutic benefit of PARPis is not durable, with acquired resistance occurring in 40% to 70% of patients [41] (Fig. 3). This is mainly due to the fact that tumor cells are prone to acquire resistance to PARPis after prolonged exposure, limiting its long-term clinical use. In recent years, several potential mechanisms of acquired resistance to PARP inhibitors are known [50]. For example, restoration of HR repair [51], reestablishment of replication fork stability [52], and drug efflux [53]. In cancers associated with germline BRCA mutations, acquired resistance to PARPis often accompanied by secondary mutations that restore BRCA function. In addition, acquired deletion of RAD 51 promoter methylation [54], mutations in Pax2 trans-activating structural domain interacting protein (PTIP) [55] or REV 7 [56], loss of PARG, and loss of 53BP1 lead to a shift in DNA repair balance from NHEJ to HR [57, 58], thereby restoring HR repair, diminishing the efficacy and eliminating synthetic lethality of PARP. The strategies to overcome the drug resistance of PARP inhibitors mainly include: combined chemotherapy, targeting the vulnerabilities related to drug resistance of PARP inhibitors, inhibiting genomic instability, and delaying the development of drug resistance by increasing the anti-tumor effect of PARP inhibitors. Current methods focus on the use of PARP inhibitors in combination with other DNA damage response inhibitors, immune checkpoint inhibitors or targeted therapies to deal with drug resistance [59].

### ATR and ATM inhibitors

After being activated by DNA single-strand breaks or replication stress (RS), one of the key kinases in the process of repairing DNA damage is the ataxia telangiectasia mutant gene Rad 3-related (ATR) kinase [60, 61]. This in turn triggers the ATR-CHK1 signaling cascade, leading to cell cycle arrest in G2-M phase and repair of DNA double-strand breaks, thereby escaping apoptosis [62, 63]. Cancer cells, particularly those with defects in ATM/CHK2/p53, often rely on ATR signaling to maintain DNA replication. Inhibiting ATR activity impairs not only the DNA checkpoint response but also the overall mechanism of double-strand break (DSB) repair, making ATR an attractive target for cancer therapy [11]. ATRi induces DNA damage in a replication-dependent and MUS81 nuclease-dependent manner, triggering the cGAS pathway, which exerts a synthetic lethal effect on MMR-d cells [64]. It was confirmed that deletion of RASSF1A expression sensitized oesophageal cancer cell lines to ATRi (e.g. VE-822) both in vitro and in vivo [65]. Thus, ATR inhibitors can selectively kill cancer cells while preserving normal cells with intact DDR. Several ATR inhibitors are currently undergoing phase I/II clinical trials, demonstrating their potential both as monotherapies and in combination with other treatments, especially in enhancing radiosensitivity and in combination with immunotherapy [66]. Studies indicate that the combination of ATR inhibitors and immune checkpoint inhibitors (such as PDL-1) shows synergistic anti-tumor activity in cancers like castration-resistant prostate cancer [67]. Additionally, ATR inhibitors have also shown promising efficacy in combination with chemotherapy agents (e.g., platinum-based drugs, topoisomerase inhibitors, PARPis, etc.), particularly in the treatment of solid tumors, highlighting their substantial clinical potential [68].

Ataxia telangiectasia mutated (ATM) kinase is an important regulator of DDR and is essential for homologous recombination repair (HRR) to restore replication fork topology [69]. ATM is activated by DNA DSBs through the MRN complex (MRE11/RAD50/NBS1), which phosphorylates and activates checkpoint kinase 2 (CHK2), G1-S, preventing entry into the S phase. thereby providing time for DNA damage repair [70–72]. In TP53 or ATM-deficient CLL cells, cancer cells are dependent on the remaining ATR-CHK1 pathway for survival, and inhibition of ATR signaling by ATRi (AZD6738) leads to accumulation of unrepaired DNA damage and eventual death due to mitotic catastrophe [73, 74]. Factors affecting ATRi sensitivity have been identified in single-agent therapy, with ATM deletion being the most significant [75]. We found that potential synthetic lethal chaperones that have been identified for ATRi targeting also include genomic ARID1A and HRR defects [24, 76]. In addition, it has been found that DNA-PKcs plays a key role in repairing double strand breaks (DSBs) through non-homologous end junctions (NHEJ) in the cell cycle, and its deletion makes cells sensitive to DSB inducers [77–79]. The substrate phosphorylation of ATM and DNA-PKcs in the treatment of DSBs is partially redundant, so there is a synthetic lethal relationship between DNA-PKcs and ATM. DNA-PKcs inhibitors may be effective in the treatment of ATM deficient tumors. It also provides a new idea for the treatment of ATM defects [80, 81]. XRD-0394, as a dual inhibitor of ATM and DNA-PKcs, has entered the phase I clinical trial, but it is still under further verification [82]. Although there are currently no specifically approved drug formulations for ATM, five promising ATM inhibitors have entered clinical development. These inhibitors—M3541, AZD0156, AZD-1390, Lartesertib (M4076), and XRD-0394—are currently undergoing Phase I clinical trials (Table 1). Their focus is primarily on treating solid tumors, particularly recurrent glioblastoma multiforme, metastatic brain cancer, and head and neck squamous cell carcinoma.

### WEE1 inhibitors

Tyrosine kinase (WEE1) is a key protein kinase expressed during the replication stress (RS) response that functions as a negative regulator of the G2-M cell cycle checkpoint [83, 84]. When the ATR signaling pathway is activated, CHK1 activation phosphorylates WEE1, inactivating the cell cycle protein-dependent kinases CDK1 (Tyr15) and CDK2, and the cell cycle arrests in the G2/M phase [85, 86]. CHK1, a highly conserved serine/threonine kinase, is located downstream of the ATR and ATM in the DDR cascade, and CHK1 inactivates the cytokinesis cycle 25 (CDC25c), thereby inhibiting CDK activity and allowing time for DNA repair [87]. The use of WEE1i in cancer therapy blocks cancer cells from using WEE1 for DNA damage repair, and cancer cells enter mitosis prematurely, increasing DNA DSBs and ultimately leading to cancer cell death [62].

Studies have shown that the WEE1 inhibitor Adavosertib (AZD1775) has a significant synthetic lethal effect on tumors carrying p53 mutations, and the drug has been shown to be effective against a wide range of TP53-mutated cancers including colorectal [88], gallbladder [89], breast [90], pancreatic [91], and prostate cancers [92]. The lack of a functional G1/S cell cycle checkpoint in TP53-deficient tumors tends to be overdependence on the G2/M checkpoint, in which case inhibition of key regulatory proteins in the G2/M checkpoint, such as WEE1, directs tumor cells into apoptosis or increases sensitivity to other therapeutic approaches by blocking the cell cycle [93–95]. In addition, inhibitors of PKMYT1 (membrane-associated tyrosine- and threonine-specific CDC2 inhibitory kinase), ano ther member of the WEE kinase family, have shown significant effects in TP53-deficient cells [96]. This was further confirmed in a genome-wide CRISPR-CAS9 screen by Toledo et al. [97]. However, single-agent therapy is prone to drug resistance, and combination drug therapy such as WEE1 and PKMYT1 inhibitors are more effective than single-agent therapy in ovarian cancer [98, 99]. The combination of WEE1 and CHK1 inhibitors demonstrated a synergistic inhibitory effect on the growth of B and T cell precursor acute lymphoblastic leukaemia (B/T-ALL) tumors and induced tumor cells to enter apoptosis [100]. However, the sensitivity of WEE1i was not affected by tumor homologous recombination repair (HRR) defects and may be associated with a complex of biomarkers of early entry into the S phase, including TP53 mutations, RB1 mutations, and CCND1 amplification [101]. In order to improve the efficacy of anticancer therapy, combinatorial drug treatment strategies should be further explored and optimized, especially for tumors with specific gene mutations.

### PRMT inhibitors

**Fig. 4:**
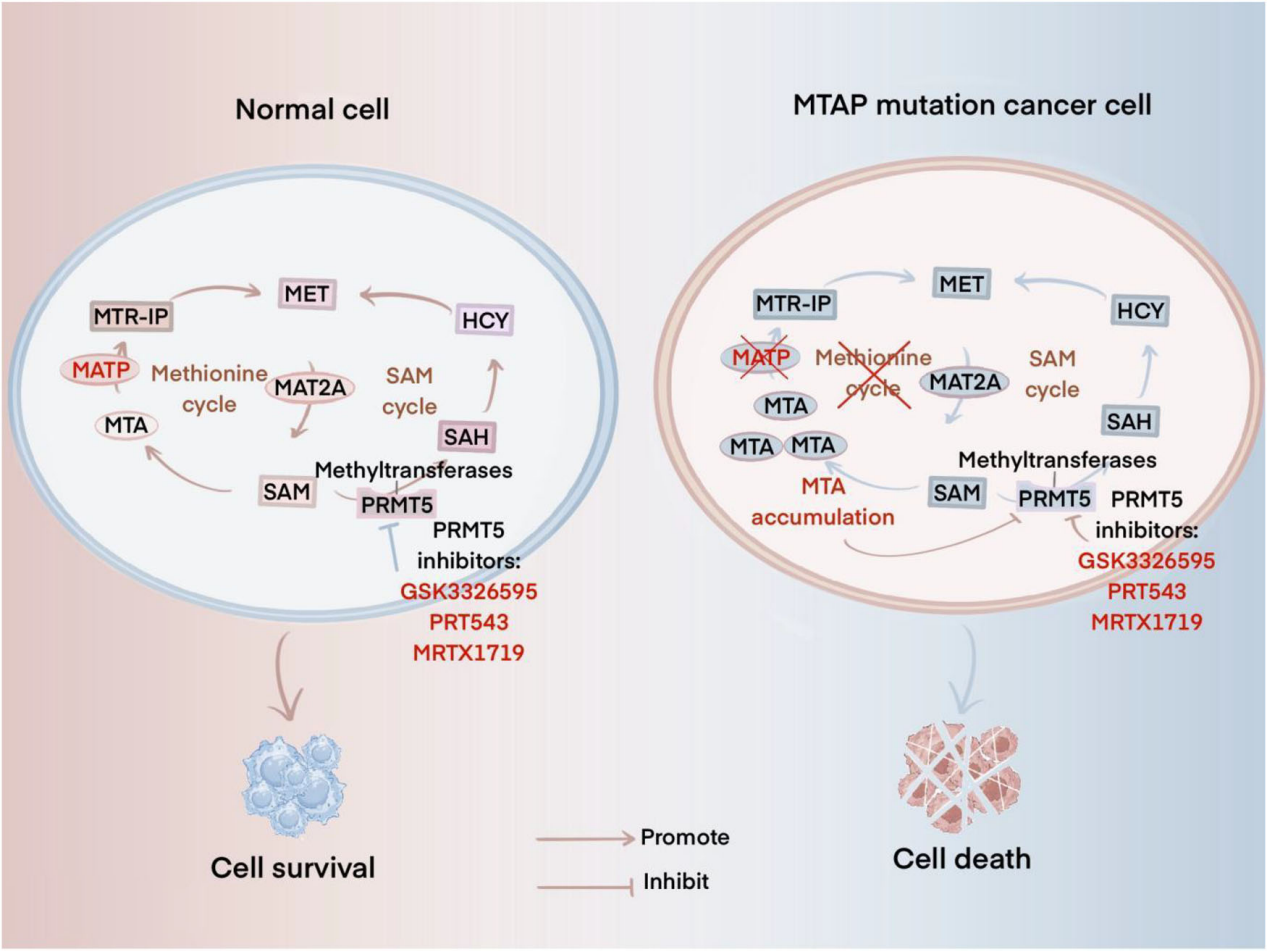
Mechanism of synthetic lethal interaction between PRMT5 and MTAP. (Left) In normal cells, MTAP is active and converts MTA to SAM through the methionine cycle. SAM, as the substrate of PRMT5, produces SAH by methyltransferase. The use of PRMT5 inhibitors can relatively reduce the methylation process, but will not affect cell survival. (Right) In MTAP-mutated cancer cells, MTAP loses its activity, causing MTA to accumulate in the cell and compete with SAM, which inhibits the activity of PRMT5 and makes the cells more sensitive to PRMT5 depletion. If PRMT5 inhibitors are used, the cells will die.SAM, S-adenosyl methionine; MTAP, methylthioadenosine phosphorylase.

The PRMT family is a group of enzymes that play a key role in cellular processes and are responsible for catalyzing the methylation of arginine [102]. In the human body, a total of nine protein arginine methyltransferases (PRMT) exist [103]. PRMT5 functions primarily as a negative regulator of transcription, and its absence leads to cell cycle arrest and apoptosis due to massive dysregulation of cellular splicing [104]. Kryukov et al. [105] found that inhibition of PRMT5 selectively kills mutation-carrying cancer cells, and is particularly sensitive to tumors deficient in methylthioadenosine phosphorylase (MTAP). Loss of MTAP leads to the accumulation of its substrate, methylthio adenosine (MTA) accumulation, which occupies the S-adenosylmethionine (SAM) binding pocket, leading to the down-regulation of PRMT5-mediated symmetric dimethylation of proteins in the cell, decreasing PRMT5 activity and leading to an increased susceptibility to PRMT5 depletion [106, 107] (Fig. 4). In addition to this, inhibition of PRMT5 leads to DNA damage accumulation and induction of the p53 response, suggesting its potential role in cancer cell therapy [108]. We also found that in ovarian cancer and non-small cell lung cancer, PRMT5 inhibition in combination with PARP inhibitors acted synergistically, with a stronger killing effect on tumor cells and even re-sensitizing resistant ovarian cancer cells to PARP inhibitors [109].

However, the results of monotherapy clinical trials of two PRMT5 inhibitors, PRT543 (NCT03886831) and GSK3326595 (NCT04676516), are not ideal, and only a few patients get a good response [103]. Recent studies have found that regulating the activity of MAT2A may provide a new strategy for the treatment of MTAP deletion cancer. MAT2A indirectly enhances the methylation of PRMT5 by promoting the production of SAM, which is closely related to the tumor progression of liver cancer [110] and colorectal cancer [111, 112]. MAT2A inhibitors, such as SCR-7952, show strong and selective anti-tumor activity in both in vitro and in vivo models of MTAP-deficient cancer, and can produce synergistic antitumor effects when used competitively or cooperatively with S-AdenosylMethionine and PRMT 5 inhibitors [113]. In addition, another small molecule MAT2A inhibitor (IDE397) is currently in phase I clinical trials for MTAP-negative solid tumors (NCT04794699) [103]. Therefore, MAT2A inhibitors have the potential to be used as therapeutic candidates for MTAP deficient cancers.

## Synthetic lethal emerging therapies and new targets

In recent years, with the progress of DNA damage response (DDR) research, a number of new synthetic lethal targets have been found, and related small molecule inhibitors have shown good potential in preclinical practice. Including DNA-PKcs, CHK1, CDK12, RAD51, POLθ and WRN, inhibitors of these pathways can provide new opportunities for cancer treatment by inducing synthetic lethality. As the core component of NHEJ, DNA-PK inhibition can enhance cytotoxicity when combined with DSB-induced radiotherapy or chemotherapy, showing a new opportunity for anticancer therapy [114]. It is worth noting that defects in both ATM and DNA-PKcs can lead to synthetic death, suggesting that DNA-PKcs inhibitors may also be promising for the treatment of tumors with ATM mutations [115]. The high expression of RAD51 is closely related to the resistance of tumors to traditional therapy, especially the interaction between RAD51 and BRCA2 and the synthetic lethal cooperation with PARP1/2, which further enhance its potential as a therapeutic target [116]. At present, RAD51 inhibitor CYT-0851 has been tested in phase I/II clinical trials, showing selective cytotoxic activity against chronic lymphoblastic leukemia mouse model and human cancer cells, and has the potential to treat B-cell hematological malignant tumors and advanced solid tumors when used alone or in combination with chemotherapy. In addition, driven by large-scale CRISPR/Cas9 knockout and RNA interference (RNAi) silencing large-scale functional screening studies, it was found that WRN inhibitor (HRO761) induced double-stranded DNA break (DSB) and activated DDR to induce WRN degradation in MSI cells and selectively promote apoptosis and cell cycle arrest [117]. These findings suggest that the deletion of WRN helicase causes synthetic lethality in microsatellite unstable (MSI) tumor models, especially in hereditary nonpolyposis colon cancer [118, 119]. In general, small molecule inhibitors targeting these emerging synthetic lethal targets are not only expected to improve the accuracy of cancer treatment, but also overcome drug resistance of PARP inhibitors and provide new treatment options for cancer patients.

## New mechanism of synthetic lethality: activation/over-activation of the conditional pathway

**Fig. 5:**
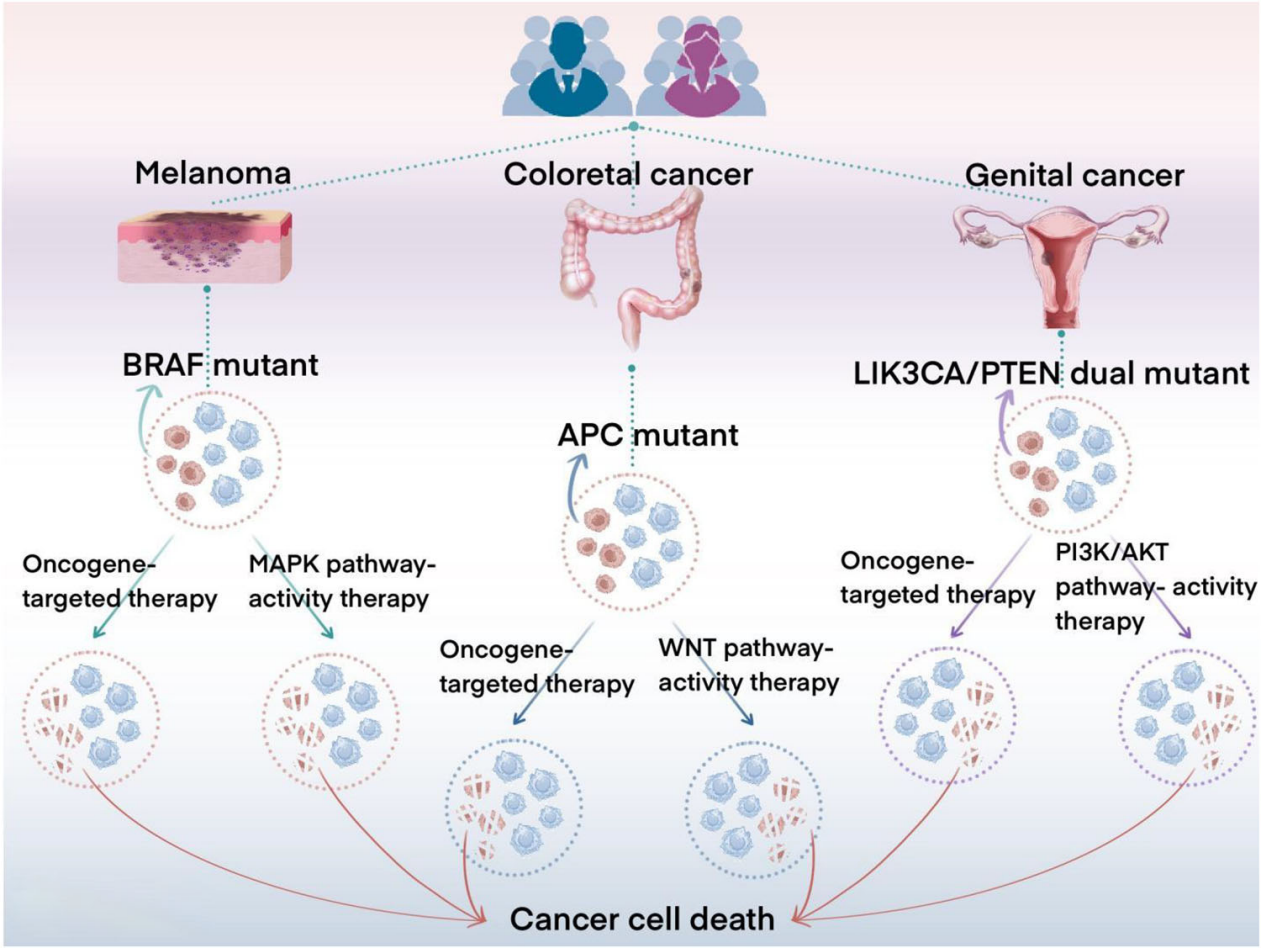
Relationship between carcinogenic pathway activity and cell death. In cancer models with specific driving mutations (such as MAPK, PI3K, WNT), the elevation of background signals makes them easy to lose their viability due to the further activation of signal pathways. Cancer cells achieve the best adaptation in the active environment of moderate carcinogenic pathway caused by these mutations. Traditional oncogene targeting therapy reduces the health level of cancer cells by reducing the activity of carcinogenic pathways. Inspired by this, a new treatment may also reduce the health of cancer cells by increasing the activity of these pathways.

Chang et al. [120] performed functionally acquired genetic perturbations in 488 cancer cell lines transduced with hundreds of DNA barcodes and systematically demonstrated that over-activation of oncogenic signaling pathways poses a lethal threat to cancer cells as well. They found that in different types of cancer cells, over-activation of different signaling pathways resulted in increased adaptive defects in the cells, which in turn inhibited cancer cell growth. For example, in some cell lines, overactivation of the MAPK pathway led to specific growth inhibition of BRAF mutant/high p-MEK level cancer cells, and hyperactivation of the PI3K pathway led to growth inhibition of endometrial cancer cells with PIK 3CA/PTEN double mutations. In addition, they found that hyperactivation of the WNT/β-catenin pathway could lead to selective death of colorectal cancer cells with APC mutations [121, 122] (Fig. 5).

### MAPK

The mitogen-activated protein kinase (MAPK) signaling pathway consists of RAF, RAS, ERK and MEK, and in conventional wisdom, low levels of MAPK are thought to inhibit cell proliferation [123, 124]. Nevertheless, resistance to BRAF and MEK kinase inhibitors is nearly always developed in BRAF (V600E) mutant melanomas [125–127]. It is now well documented that excessive MAPK activity is also toxic to cancer cells from different tissue backgrounds, and in the past, high levels of the ERK pathway were known to cause cell cycle arrest [128, 129]. However, follow-up studies have shown that very high levels of ERK activity can lead to cell death rather than cell cycle arrest [130]. Among other things, overactivation of the MEK 1/2-ERK 1/2 cascade in BRAF mutant melanoma cell lines led to significant cytotoxic effects [131]. Selective killing of drug-resistant melanoma cells is achieved by increasing intracellular reactive oxygen species (ROS) levels due to over-activated MAPK [132]. Over-activation of MAPK also induces cell death in the form of autophagy, e.g., TBMS1-induced autophagy in melanoma cells inhibits value-addition by this principle. When sustained activation of the ERK pathway is sufficient to plunge cells into autophagic vacuolization [133, 134]. In addition, to ERK, rapid hyperactivation of other oncogenic pathways such as Bcr-Abl, JAK/STAT, and NPM-ALK also triggers cell death [135].

We observed that in NPM-ALK signaling pathway-driven mesenchymal large cell lymphomas, over-activation of NPM-ALK would lead to a significant increase in phosphorylation of the DNA damage response mediated by the MAPK-ERK 1/2 pathway, resulting in the accumulation of DNA damage and γH2AX microfoci in the nucleus of the cells at the DNA double-strand breaks (DSBs), and ultimately due to the oncogenic stress response inducing impaired lymphoma growth and apoptosis [136, 137]. This implies that rapid over-activation of oncogenic signaling pathways may be a novel strategy for cancer therapy.

### PI3K/AKT

In ER-positive breast cancer, a synthetic lethal interaction between GATA 3 and MDM 2 has been identified, which acts through the PI3K/AKT/mTOR signaling pathway and is p53-dependent. Cell cycle arrest and apoptosis can be co-regulated by the PI3K/Akt/mTOR pathway and p53 under normal circumstances [138]. Nevertheless, monotherapy is insufficient to eradicate cancer cells, because PI3K inhibitors have a rather poor therapeutic index [139, 140]. Thus, the hunt for novel therapeutic modalities is about to begin.

At a later stage, there is growing evidence that overactivation of these same pathways may also disrupt cancer cell homeostasis. Over-activation of the phosphatidylinositol-3-phosphate/AKT (PI3K/AKT) signaling pathway promotes oxidative metabolic pathways in chronic lymphocytic leukaemia (CLL) cells, with excessive accumulation of ROS mediating dependent cell death. This is despite the fact that, contrary to conventional wisdom, it is believed that AKT overexpression promotes only cell proliferation and survival in CLL [141]. Targeted therapies against the PI3K/AKT signaling pathway have become more important, and as direct activators of pharmacologically studied PI3-kinases are not available, hyperactivation of the PI3K/AKT signaling pathway was achieved by knockdown or inhibition of the activity of the inhibitory phosphatase SHIP 1. As a result, activation was significantly increased by SHIP-1 inhibition, and AKT phosphorylation was expressed at some of the highest baseline levels [142].

### WNT

The majority of colorectal cancer CRCs exhibit genetic changes in the WNT pathway, such as APC mutations, activating mutations in CTNNB1 (β-catenin), etc., which could encourage the initial growth of the tumor [143–145]. Certain inhibitors of the WNT/β-catenin signaling pathway have emerged as potential cancer therapeutic agents, and down-regulation of WNT signaling can inhibit cancer progression [146]. For example, the discovery of the natural compounds CPG049090, PKF115-584 and PKF222-815 has attracted much attention. They were confirmed to interfere with the interaction of the β-connexin/TCF complex, and one of them, the inhibitor PKF115-584, blocked β-connexin-dependent transcriptional activity, which was demonstrated to inhibit cancer cell proliferation in in vitro assays in colon cancer cells [147, 148]. However, cancer cells can acquire resistance to anticancer drugs by maintaining their genomic stability through DNA damage repair pathways to avoid apoptosis [149].

It was found that forced hyperactivation of the same oncogenic pathway can also selectively destroy the viability of cancer cells, and instead of promoting cancer development, hyperactivation showed anti-cancer activity [150, 151]. In CRC cells, histone deacetylase (HDACi) inhibitors can mediate the overactivation of the WNT signaling pathway to inhibit cell proliferation and induce apoptosis in colorectal cancer cells [152]. Colorectal cancer (CRC) subpopulations with mutations in the tumor suppressor gene APC are more likely to be inactivated by overexpression of β-catenin. In addition to overexpression of β-catenin, knocking down the APC gene in APC-mutant CRC also leads to tumor cell death [121]. The most common genetic alterations of the negative regulator APC in this pathway are allelic deletions or loss-of-function mutations [143]. In tumor samples, elevated expression of classical CTNNB1-dependent transcriptional WNT targets correlates with elevated CTNNB1 protein levels, suggesting that CTNNB1 stabilization is consistent with WNT-dependent transcriptional hyperactivation [153]. In addition, hyperactivation of the WNT signaling pathway synergistically interacts with Rb inactivation, leading to an increase in mTOR activity, which induces apoptosis and causes energy stress and oxidative stress [154].

In addition, there are many SL potential targets to be explored. CIP2A is a crucial gene in BRCA1- and BRCA2-mutant cells, according to Daniel Durocher et al.‘s research on a genome-scale CRISPR-Cas9 synthetic lethality screen in isogenic pairs of BRCA1- and BRCA2-deficient cells. The physical disruption of the complex between cip2a and topbp1 is particularly deleterious in tumors lacking BRCA, indicating that CIP2A could be a viable therapeutic target for BRCA1 and BRCA2 mutant malignancies [155]. Stephen J et al. searched for alternative targets of synthetic lethality in the context of BRCA2 gene defects and screened two pairs of BRCA2 gene homozygous cell lines with shRNAs focused on DNA repair and CRISPR. The genetic screen identified FEN1 and APEX2 as BRCA2’s artificially lethal targets [156]. FEN1 nucleic acid endonuclease was identified by Kolodner RD et al. as a therapeutic target for homologous recombination-deficient human cancers. Additionally, small-molecule FEN1 inhibitors and small-interfering RNAs of FEN1 selectively killed human cell lines deficient in BRCA1 and BRCA2, confirming the sensitivity of these cancers to FEN1 deficiency [157]. Hinze et al. [158] discovered a synthetic lethal interaction between Wnt pathway activation and asparaginase in acute leukemia resistant to the enzyme using a genome-wide CRISPR/Cas9 screen. While asparaginase sensitivity was not enhanced in normal hematopoietic progenitor cells, it was in various drug-resistant subtypes of acute leukemia due to Wnt pathway activation. Asparaginase treatment was synthetically lethal in acute leukaemia, activating Wnt-dependent stabilization of proteins and thereby reducing GSK3-dependent protein degradation. Inhibition of GSK3α sensitizes asparaginase-resistant leukaemia to treatment.

## Status of clinical studies on synthetic lethality

A third or so of SL clinical trials deviate from the widely researched DNA damage response (DDR) pathway, indicating the growing SL usage in clinical oncology. Compared to non-SL trials, SL oncology trials have a greater success rate. Nevertheless, about 75% of SL interactions that have been preclinically verified have not yet been investigated in clinical trials [159].

### Specific applications of current synthetic lethal strategies

The number of SL-based clinical trials has grown annually since the initial trials evaluating the effectiveness of PARP inhibitors for the treatment of patients with BRCA-mutated breast cancer. In an effort to provide a comprehensive picture of SL oncology clinical trials, researchers examined Trialtrove, a for-profit clinical trial database. They discovered that roughly one-third of SL clinical trials extend beyond the usual focus of study, indicating the growing trend of SL use in clinical oncology.

In terms of number of SL studies, the top five cancers are lymphoma, colorectal, lung, ovarian, and breast cancer. It is interesting to note that DDR route drives the SL studies in the majority of solid tumors, including ovarian, lung, and breast cancer, whereas non-DDR pathways drive the SL trials in blood malignancies, like lymphoma and chronic lymphocytic leukemia [159].

In SL studies, patient histology data need to be obtained and analyzed in a timely manner to standardize heterogeneity in the histology data. Integrated analysis of multi-omics data, such as RNA-seq, histology images, DNA-seq, proteomics, methylation, etc., is also particularly important, and combining histological data with clinical data is expected to significantly improve the contribution of SL to precision oncology [160–162].

Based on statistical inference, researchers have developed Synthetic Lethality Identification (SiLi), a novel approach to target liver cancer. This method allowed for the identification of SL interactions in liver cancer and the identification of 272 SL pairings, comprising 209 distinct potential targets. Polo-like kinase 1 (PLK1) is one of them and is thought to have a lot of therapeutic promise. Additional computational and experimental verification of SL pairing TP53-PLK1 implies that PLK1 inhibition might be a potential treatment approach aimed specifically at individuals with liver cancers that are TP53-mutant [163].

### Network screening and prediction in synthetic lethal strategies

Using a genetic screen, the study team discovered that the Werner syndrome RecQ deconjugase WRN is a synthetic lethal target in microsatellite instability (MSI) cancer cells. A strong, particular WRN inhibitor is HRO761.The phenotypes shown with WRN gene inhibition were recapitulated by pharmacological inhibition of HRO761, which resulted in tumor development and DNA damage in MSI cells while selectively inhibiting cell proliferation. Researchers saw dose-dependent in vivo production of DNA damage and suppression of tumor development in MSI cells and patient-derived xenograft models with oral HRO761 therapy. These results show that WRN is a viable therapeutic target for MSI cancer based on preclinical pharmacological validation. The safety, tolerability, and preliminary anti-tumor activity of HRO761 in patients with MSI solid tumors, including colorectal cancer, are presently being assessed in a clinical trial (NCT05838768).

The study also mentioned the possible synergistic effect of the combination therapy of HRO761 with irinotecan, a topoisomerase I inhibitor, which provides new ideas for the treatment of MSI cancer patients. The findings not only provide pharmacological validation of the synthetic lethality of WRN in cancer, but also provide important information for the development of new cancer treatment strategies [117].

Computational prediction is a vital tool for expediting SL screening, however, present methods are subject to selection bias in SL data and are dependent on cancer- and tissue-specific histology. The scientists developed ELISL (Early-Late Integrated SL prediction with forest ensembles), which demonstrated promising cross-cancer predictions, excellent resilience to selection bias, and recovery of known SL genes across several cancer types. Longer patient survival was linked to ELISL prediction of concurrent mutations from the HH, FGF, WNT, or NEIL gene families as well as the BRCA gene, suggesting possibilities for therapy [164]. The most reliable model under changing gene selection bias was the ELISL model, which outperformed other SL prediction techniques. Still, learning from skewed data is a fundamental machine learning problem that needs more investigation.

Based on the concept of synthetic lethality, researchers created the Synthetic Lethality Knowledge Graph web server (SLKG, http://www.slkg.net) to investigate holistic oncology treatment perspectives and identify cancer-specific susceptibility. The computational foundation for identifying treatment approaches based on the concept of synthetic lethality and specifically tailored to cancer is provided by SLKG [165].

Li Guo et al. integrated multi-omics data (primarily DNA mutations, copy number variations, methylation, and mRNA expression data) to analyze candidate genes based on a set of public gene pairs with synthetic lethal interactions. They then used random forests to predict synthetic lethal interactions that are specific to cancer. The SLOAD database, a user-friendly database for browsing, searching, downloading, and analyzing data, was created by integrating these discoveries in their ultimate form [166]. This project may yield candidate synthetic lethal interactions that are particular to cancer, which may aid in the development of drugs for cancer treatment and encourage the use of synthetic lethal principles in therapeutic approaches.

## Potential in synthetic lethality research and the technical challenges

The technical challenges of synthetic lethality have been a topic of interest in various research fields. Identification of synthetic lethal partner genes under various conditions remains a challenge [58]. The search for these gene pairs is arduous and fraught with technical challenges, making it a complex task. Moreover, the use of synthetic lethality for therapeutic purposes faces a number of technical challenges, such as (1) Determining which gene combinations have synthetic lethal potential requires in-depth genomic and bioinformatics analyses. (2) Discovery and validation of biomarkers that can predict a patient’s response to synthetic lethality therapy is needed [167]. (3) Drug development as well as drug delivery is also one of the challenges, developing small molecule drugs or biologics that can specifically target these gene combinations while avoiding toxicity to normal cells. (4) The challenge of drug resistance is also considered in synthetic lethal therapy, as tumor cells may develop resistance to therapeutic agents and strategies need to be developed to overcome or prevent resistance. (5) In the course of treatment, synthetic lethal therapies may need to be used in combination with other therapeutic approaches (e.g., radiotherapy, chemotherapy, or immunotherapy), which adds to the complexity of treatment strategies. And there is tumor heterogeneity between different tumor types: cells within a tumor may have different genotypes and phenotypes, which may affect the efficacy of synthetic lethal therapy.

Differences in genetic and/or epigenetic background in individual cells may explain the cell environment-dependent synthetic lethality observed in various studies. Since a single tumor may carry an average of 30-70 mutated genes [168], a single primary tumor or established cancer cell line may carry multiple simultaneously activated oncogenes or inactivated oncogenes. Activation of other signaling pathways may provide redundant inputs that drive and maintain downstream survival signals, leading to resistance to therapeutic agents that directly or indirectly through synthetic lethality target specific genetic lesions. In addition, even though mutations in certain gene vectors in tumor cells may not lead to tumorigenesis, these mutations may affect the response to drugs due to alterations in drug metabolism or drug efflux [169].

Interdisciplinary collaboration can drive further development of synthetic lethality research, which requires the collaboration of multidisciplinary teams of molecular biologists, pharmacologists, clinicians, and bioinformaticians. Interpreting useful information from large amounts of genomic data and translating it into clinical applications requires complex data analysis and model building.

## Synthetic lethal drug development

Several different dual-target drug strategies, including targeting synthetic lethality, compensatory mechanisms, mutational resistance, synergistic effects, and other emerging strategies are feasible in clinical practice. For example, some drugs have been designed to simultaneously inhibit EGFR and ALK, two mutant kinases commonly found in NSCLC, as a way to overcome the resistance of tumor cells to single-target EGFR inhibitors [170]. In addition, drugs have been developed to simultaneously inhibit two signaling pathways that compensate for each other in tumor cells as a way to enhance the therapeutic effect [171].

Efforts to use SL to identify predictive biomarkers, new therapeutic targets and combination therapies for effective populations reinforce the hope that SL may become a key driver of precision oncology in the future. Since 2016, a wave of biotech companies utilizing synthetic lethality have emerged, with research focusing on the discovery of synthetic lethal drugs for metabolic targets in cancer, personalized synthetic lethal drugs for the tumor microenvironment and tumor immunology research, DNA damage repair inhibitors, the discovery of synthetic lethals using CRISPR and more, respectively.

KSQ Biotech has generated a large dataset by knocking out the entire genome of nearly 600 cell lines one by one, and the extensive screening has helped it understand whether a drug can be safely targeted.

Nevertheless, the safety of the strategy is one of the draws of combining checkpoint inhibitors with DNA repair inhibitors. Early clinical results indicate that the combination of immunotherapy and PARP inhibitors does not worsen adverse effects, in contrast to adding a kinase inhibitor, which has been demonstrated to be harmful in certain situations.

Companies working on developing synthetic deadly drugs are becoming more and more numerous, and as a result, they are thinking about how to modify their medication candidates for the quickly changing oncology market. As cancer immunotherapy has grown in importance, it will be crucial to think about how to mix its DNA repair inhibitors with other immunotherapies that have been licensed, such as checkpoint inhibitors and other immuno-oncology medications, in the future. Although it makes sense in theory to combine DNA repair inhibitors with immunotherapies, it can be difficult to investigate these combinations in the lab. We are currently dealing with a lack of data. Furthermore, there is a great deal of unanswered research regarding the potential synergy between DNA repair and immunotherapy because the immune system is hard to simulate in cells and animals. Interdisciplinary collaboration to advance tumor synthetic lethality research, as well as related drug development, is also particularly important.

## Predicting the future of synthetic lethal strategies in cancer treatment

Synthetic lethal strategies (SL) show great potential in cancer therapy. With advances in biotechnology and a deeper understanding of tumor heterogeneity, SL is expected to improve tumor specificity, reduce therapeutic toxicity and improve patient prognosis. The identification of genetic and functional biomarkers is critical to determining the patient population most suitable for receiving this therapy. However, there are still many challenges in moving from drug target discovery to clinical application, with drug resistance being a major challenge. Cancer cells can develop drug resistance through genetic mutations, reprogramming of signaling pathways, and altering the expression levels of drug targets. To address these challenges, cancer cells have been found to be sensitive to over-activation of the MAPK/PI3K/WNT pathway, but the molecular mechanisms of oncogenic pathway over-activation-mediated anti-tumor effects have not been fully elucidated, and this approach has not yet been extensively studied. Further in-depth studies on the mechanisms of conditional activation are needed to explore how to effectively achieve forced hyperactivation of signaling pathways in order to precisely intervene in cancer cells without affecting normal cells.

In the past decades, the popularity of targeted drugs has achieved significant success in cancer treatment [172]. However, due to the diversity of tumor pathogenesis and the complexity of the tumor microenvironment, single-targeted drugs cannot achieve the desired effect in many cases, which in turn results in tumor immune escape. Moreover, in the process of using targeted drugs, problems such as tumor compensation and targeted drug resistance also emerge. The combination of single-targeted drugs may cause various clinical adverse effects, as well as pharmacokinetic interactions, so dual-targeted drugs are attracting the attention of researchers. Dual-target drugs utilize predictable bioavailability, pharmacokinetics and metabolism without the risk of drug interactions. It is reassuring that the emergence of more and more new and emerging technologies may facilitate the development of dual-target drugs. Therefore, we believe that synthetic lethal therapeutic strategies for these dual-target drugs will help provide more new small molecule drug candidates for future cancer treatment.

Synthetic lethal drug target discovery has been made possible by CRISPR technology, which directs the Cas9 enzyme to precisely localize to specific regions of the genome by using a guide RNA 20 base pairs in length to achieve DNA cleavage that results in the loss of function of a specific gene product [173–175]. The application of this approach in human cells has allowed us to systematically study the impact of the loss of each gene on cellular function. Since the first discovery of synthetic lethality in Drosophila, many cancer patients have been cured, and the application of the genome editing capabilities of CRISPR technology heralds the hope of cures for many more in the future [12]. CRISPR/Cas9 gene editing technology, libraries of DNA-encoded compounds, and the use of artificial intelligence in drug design. The application of these technologies is expected to increase the efficiency of discovering novel synthetic lethal drugs.

Future targeted cancer therapy strategies may become more diverse, not only limited to inhibiting oncogenic signaling pathways, for example, but also exploiting their hyperactivation for therapeutic purposes. However, clinical data is still limited, and this area still requires intensive research. Through continued scientific exploration and clinical trials, the synthetic lethal strategy is expected to become a more effective and personalized cancer treatment in the future.

## Data Availability

Not applicable.
